# Commentary on “Low Thoracic Muscle Mass: A Novel Indicator of Poor Prognosis in Patients With Severe Fever With Thrombocytopenia Syndrome”

**DOI:** 10.1002/iid3.70509

**Published:** 2026-09-08

**Authors:** Mohammad Barary, Mostafa Javanian, Mohammad Golparvar Azizi, Mehran Shokri, Soheil Ebrahimpour

**Affiliations:** ^1^ Student Research Committee, Department of Pharmacology School of Medicine, Shahid Beheshti University of Medical Sciences Tehran Iran; ^2^ Infectious Diseases and Tropical Medicine Research Center Health Research Institute, Babol University of Medical Sciences Babol Iran; ^3^ Department of Internal Medicine School of Medicine, Rohani Hospital, Babol University of Medical Sciences Babol Iran

Dear editor,

We read with great interest the article by Wang et al. entitled “Low Thoracic Muscle Mass: A Novel Indicator of Poor Prognosis in Patients with Severe Fever with Thrombocytopenia Syndrome,” currently published in your esteemed journal [1]. The purpose of the present research was to identify and analyze the clinical features and prognosis of patients with severe fever with thrombocytopenia syndrome (SFTS) who had normal or low skeletal muscle index (SMI). The findings of this investigation disclose that low SMI is associated with adverse outcomes in patients with SFTS. On the other hand, SMI may serve as a reliable prognostic marker for SFTS in clinical settings. While these insights contribute valuable knowledge to the domain, some interpretive issues limit the robustness and applicability of the conclusions.

First, the retrospective design increases concerns about selection bias and external validity. We would suggest future replication in prospective, multicenter cohorts to confirm these associations and to support broader practice changes. Furthermore, the time frame (2020–2023) overlaps the COVID‐19 era, during which healthcare utilization, testing availability, and clinical decision‐making evolved. The study would benefit from a transparent discussion of how pandemic‐era factors were accounted for (e.g., changes in hospital discharge criteria and ICU admission thresholds).

Second, the analysis adjusted for age and sex, but vital social determinants and comorbidity variables appear to be under‐ or unmeasured. The important factors such as socioeconomic status, occupational status, educational level, rural versus urban residency, insulin resistance, nutritional status, physical activity, waist circumference, body mass index (BMI), psychosocial stress, detailed smoking status (current, former, heavy smokers), alcohol consumption (mild, moderate, severe), and opioids consumption, and broader comorbidity burden (e.g., dyslipidemia) might influence on outcomes. The respected authors should consider expanding covariate adjustment or providing a robust discussion of residual confounding [2, 3].

Third, the absence of relevant laboratory parameters and biomarkers decreases the overall completeness of the study's conclusions. Incorporation of further biochemical markers, including ferritin, hemoglobin A1c (HbA1c), erythrocyte sedimentation rate (ESR), high‐sensitivity C‐reactive protein (hsCRP), serum levels of vitamin C, D, E, magnesium, zinc, selenium, and inflammatory ratios (e.g., Neutrophil‐to‐Lymphocyte RatioN, Platelet–to‐Lymphocyte Ratio, Monocyte‐to‐Lymphocyte Ratio, Systemic Immune‐Inflammation Index) might illuminate biological pathways underpinning mechanistic inferences [4, 5].

Fourth, this investigation would benefit from enhanced transparency regarding pharmacotherapy, including explicit detailing of concomitant medications, dosing regimens, and changes in therapy over the study period. Data on the quality of antidiabetic therapy, antihypertensive drugs, lipid‐lowering medications, and other relevant medications may influence outcomes [6]. Moreover, the endpoint is death or discharge, which blends mortality with discharge decisions that may reflect nonmortality outcomes. If possible, mortality should be the primary endpoint, with discharge timing analyzed as a secondary outcome. It would be prudent to clarify whether discharge is treated as a death‐equivalent endpoint or whether discharge causes are primarily attributable to clinical improvement or patient transfer. Also, several tables present overlapping information with inconsistent formatting. The clear separation of baseline characteristics, imaging metrics, and outcomes would improve readability.

In summary, although the recent study offers considerable insight into a novel indicator of poor prognosis in patients with SFTS, addressing the methodological limitations identified above would substantially strengthen the reliability and translational relevance of the conclusions. We respectfully urge the authors and the editorial board to consider these points and provide additional clarification.

## Author Contributions

**Mohammad Barary:** investigation, writing – original draft, writing – review and editing. **Mostafa Javanian:** investigation, writing – original draft. **Mohammad Golparvar Azizi:** investigation, writing – original draft. **Mehran Shokri:** writing – original draft, investigation. **Soheil Ebrahimpour:** investigation, writing – original draft, supervision. All authors read and approved the final manuscript.

## Funding

The authors have nothing to report.

## Ethics Statement

The authors have nothing to report.

## Consent

The authors have nothing to report.

## Conflicts of Interest

The authors declare no conflicts of interest.

## Data Availability

Data sharing not applicable to this article as no datasets were generated or analyzed during the current study. The authors have nothing to report.
